# Nonlinear Redox–Immune Coupling Under Low-Dose-Rate Radiation: A Compartment-Specific Framework for Biological Responses—A Narrative Review

**DOI:** 10.3390/antiox15060782

**Published:** 2026-06-22

**Authors:** Dawon Kang

**Affiliations:** 1 Department of Physiology, College of Medicine, Gyeongsang National University, Jinju 52727, Republic of Korea; dawon@gnu.ac.kr; Tel.: +82-55-772-8044; 2 Department of Convergence Medical Science, Gyeongsang National University, Jinju 52727, Republic of Korea; 3 Institute of Medical Sciences, Gyeongsang National University, Jinju 52727, Republic of Korea

**Keywords:** low-dose-rate radiation, oxidative stress, immune modulation, inflammation, hormesis

## Abstract

Ionizing radiation induces reactive oxygen species (ROS) and inflammatory signaling that contribute to both therapeutic efficacy and normal tissue toxicity. While the effects of high-dose radiation are well characterized, responses to low-dose-rate radiation (LDRR) remain inconsistent and are not adequately explained by conventional linear dose–response models. To address this gap, we conducted a narrative review of recent experimental studies across multiple biological systems, including body fluids, joint microenvironments, and reproductive tissues, focusing on redox and immune-related responses under LDRR conditions (dose rates: 0.39–3.49 mGy/h). Literature was identified through PubMed/MEDLINE, Web of Science, and Google Scholar, with emphasis on studies published between 2015 and 2026. These studies demonstrate that LDRR elicits nonlinear, dose-dependent effects that vary across biological compartments and involve coordinated changes in oxidative stress, immune signaling, and metabolic regulation. Based on this synthesis, we propose a unifying framework of nonlinear redox–immune coupling, in which oxidative stress functions as a threshold-dependent regulator and immune responses follow a biphasic trajectory characterized by activation at lower dose rates and attenuation or adaptation at higher levels. These responses are strongly influenced by the local microenvironment, resulting in compartment-specific variability. This integrated perspective supports a shift from dose-centric to systems-level interpretations of radiation biology and provides a basis for improving biomarker development, risk assessment, and therapeutic strategies in chronic low-dose radiation exposure settings. Future research priorities include time-resolved mechanistic studies to define compartment-specific redox thresholds, validation of candidate biomarkers under identical multi-compartment experimental conditions (e.g., GSH/GSSG ratio, 8-OHdG, circulating cytokine panels including IL-10/TNF-α ratio), and integration of subject-specific biological variables (e.g., age, sex, and baseline redox capacity) into predictive models of LDRR response.

## 1. Introduction

Ionizing radiation (IR) is a cornerstone of oncology, exerting its biological effects primarily by generating reactive oxygen species (ROS) and activating cellular stress pathways [1,2,3,4,5,6,7]. While the dose-dependent cytotoxic effects of high-dose radiation are well characterized, the biological outcomes of low-dose-rate radiation (LDRR) remain poorly understood and often appear inconsistent across experimental systems [7,8,9,10,11]. This uncertainty has hindered the development of predictive models for normal tissue toxicity and limited the refinement of personalized radiotherapeutic strategies.

A fundamental challenge in radiation biology lies in the appropriate scope of linear dose–response models. The linear no-threshold (LNT) model is supported by substantial epidemiological evidence, most notably from the Life Span Study of atomic bomb survivors and the BEIR VII report, which establish a consistent association between radiation dose and stochastic cancer risk even at doses below 100 mSv [12,13]. For cancer risk estimation at the population level, LNT therefore remains the regulatory standard and is not disputed here. However, the LNT framework was not designed to predict non-stochastic, adaptive, or immune-related biological responses to chronic low-dose-rate exposure, a distinct exposure scenario characterized by concurrent damage and repair. Emerging evidence indicates that LDRR elicits nonlinear and sometimes paradoxical responses, including immune activation at lower dose rates and suppression or adaptation at higher levels [1,14,15,16,17]. It should be noted, however, that such biphasic or hormetic responses are not universally observed. Several studies have reported monotonic suppression or no measurable immune modulation under LDRR conditions, particularly at endpoints related to lymphocyte depletion and systemic immunosuppression [18,19]. These findings remain inconsistent across experimental systems, and a unifying mechanistic framework that can account for both linear and nonlinear patterns depending on endpoint and dose rate remains lacking.

Importantly, radiation-induced oxidative stress does not occur in isolation but is modulated by systemic factors, including metabolic status, microbiome composition, and environmental stressors [17,20,21,22,23,24]. These variables collectively shape redox homeostasis and downstream immune responses, contributing to variability in biological outcomes [25,26,27]. This perspective suggests that the apparent stochasticity of LDRR responses may instead reflect the integration of multiple regulatory inputs within specific microenvironments.

A critical terminological distinction must be established at the outset. Low-dose radiation (LD) refers to a cumulative absorbed dose threshold, conventionally defined as below 100 mGy, irrespective of the rate of delivery. In contrast, LDRR is defined by the rate at which dose is delivered, conventionally below 0.1 mGy/min (equivalent to 6 mGy/h), regardless of the total cumulative dose. These two concepts are independent and must not be conflated: a total dose of 1.7 Gy delivered continuously at 3.46 mGy/h over 21 days constitutes LDRR exposure despite a relatively high cumulative dose, whereas a single CT examination delivering 50 mGy represents a low dose but high-dose-rate exposure. The biological significance of dose rate lies in its relationship to cellular repair kinetics: at low dose rates, DNA repair and adaptive signaling mechanisms operate concurrently with radiation-induced damage, fundamentally altering the biological response compared to acute high-dose-rate exposure of equivalent cumulative dose [28,29]. The present review focuses specifically on dose rate as the primary biological variable within the range of 0.39–3.46 mGy/h, a range encompassing chronic occupational, environmental, and clinical low-dose-rate scenarios and well within the ICRP-defined LDRR threshold of 6 mGy/h.

Several narrative reviews have examined biological responses to low-dose radiation, focusing primarily on either oxidative stress mechanisms [30,31] or immune modulation [19,32] as largely independent phenomena. Reviews addressing dose rate effects specifically have largely been confined to epidemiological or cancer risk contexts [29,33], without integrating mechanistic redox–immune interactions. Critically, no existing review has: (1) articulated the molecular basis by which oxidative stress thresholds and immune dynamics are mechanistically coupled under LDRR conditions; (2) systematically compared biological responses across multiple tissue compartments under identical experimental conditions; or (3) translated this mechanistic framework into specific, testable biomarker hypotheses applicable to clinical LDRR scenarios. The present narrative review was undertaken to address these three gaps directly.

This review was motivated in part by the authors’ experimental program examining LDRR effects across multiple biological compartments in murine models [34,35] (Cao et al. unpublished data), which revealed consistent patterns of nonlinear, compartment-specific biological responses that lacked a unifying mechanistic explanation. These empirical observations, spanning hematological, reproductive, and peritoneal compartments, form the starting point for the integrative framework proposed here, and are positioned throughout this review as extensions of independently established findings rather than as primary evidence for the proposed model. Despite increasing recognition of the nonlinear nature of LDRR responses across biological compartments, a critical gap persists: the field lacks a unifying framework to explain how redox homeostasis and immune signaling are dynamically coupled under LDRR conditions and how this coupling varies across tissue microenvironments.

Here, we propose a unifying model of nonlinear redox–immune coupling, in which oxidative stress functions as a threshold-dependent regulator and immune responses follow a biphasic trajectory. Critically, the novelty of this framework lies in articulating how these two phenomena are mechanistically linked through shared molecular nodes, specifically, reciprocal nuclear factor erythroid 2-related factor 2 (Nrf2)/nuclear factor kappa-light-chain-enhancer of activated B cells (NF-κB) regulation and the redox sensitivity of the cyclic GMP–AMP synthase (cGAS)-stimulator of interferon genes (STING) pathway, thereby producing compartment-specific biological outcomes. This systems-level framework provides a basis for improving biomarker development, refining risk assessment, and optimizing strategies for chronic radiation exposure.

## 2. Methods

This review was conducted as a narrative synthesis of experimental evidence on LDRR-induced redox and immune responses. Electronic databases including PubMed/MEDLINE, Web of Science, and Google Scholar were searched using terms including “low dose radiation”, “low-dose-rate radiation,” “oxidative stress,” “reactive oxygen species,” “immune modulation,” “hormesis,” “Nrf2,” “NF-κB,” “cGAS-STING,” and “biological compartments.” Reference lists of identified articles were manually reviewed for additional sources. Studies were included if they reported quantitative biological outcomes following LDRR exposure in mammalian systems (in vitro, in vivo, or clinical). No restriction on publication year was applied to mechanistic or foundational studies; however, emphasis was placed on primary research published between 2015 and 2026. Studies exclusively examining high-dose radiation effects without comparison to low-dose conditions were excluded. As a narrative review, this synthesis does not follow PRISMA guidelines; selection bias is acknowledged as a limitation (see Section 9). The review type is identified as a narrative review throughout the manuscript in accordance with journal recommendations.

Throughout this review, a clear distinction is maintained between LD, defined by cumulative absorbed dose (typically <100–200 mGy), and low-dose-rate radiation (LDRR), defined by the rate of dose delivery (typically <0.1 mGy/min or <6 mGy/h) irrespective of total dose. These are not equivalent: a cumulative dose of 1.7 Gy can be delivered at a low dose rate (3.46 mGy/h over 21 days), while a low cumulative dose (e.g., 50 mGy) can be delivered at a high dose rate in a single imaging session. Studies were included in this review based on dose rate criteria, not cumulative dose thresholds. Similarly, dose rate (mGy/h) is treated throughout as the primary independent biological variable, with cumulative dose serving as a secondary, exposure-duration-dependent parameter. Additionally, chronic continuous LDRR (protracted, uninterrupted exposure with concurrent damage and repair) is distinguished from fractionated radiotherapy (discrete high-dose fractions with full repair intervals), as these represent biologically distinct exposure scenarios with different mechanisms of cellular response. Finally, findings from experimental animal or in vitro studies are explicitly distinguished from clinical interpretations throughout; experimental evidence is used to establish mechanistic plausibility, not to make direct clinical recommendations without prospective human data.

It is further acknowledged that the dose rate categorizations used in this review, low (0.39 mGy/h), intermediate (1.29 mGy/h), and high (3.46 mGy/h), are operationally defined based on the experimental range studied by the author’s group and may not correspond directly to dose rate categories used in other published studies, which have employed a wider range of LDRR values [11,29,36]. Table 1 summarizes the key experimental studies included in this review, organized by model, radiation type, dose rate, cumulative dose, exposure duration, biological system, and observed response pattern.

## 3. Oxidative Stress as a Threshold-Dependent Response

ROS generation is a central mediator of radiation-induced biological effects and represents a primary link between physical radiation exposure and downstream cellular responses [1,2,3,4]. Under high-dose conditions, excessive ROS accumulation leads to oxidative damage to lipids, proteins, and DNA, ultimately resulting in cell death. However, under LDRR conditions, ROS dynamics differ fundamentally from those observed in acute high-dose exposure, reflecting a balance between ROS production and endogenous antioxidant defenses [1,39,40,41]. At low dose rates, cells maintain redox homeostasis through tightly regulated antioxidant systems, including enzymatic pathways such as superoxide dismutase, catalase, and glutathione peroxidase, as well as non-enzymatic antioxidants [42]. In this regime, ROS act predominantly as signaling molecules that regulate cellular adaptation, metabolic activity, and stress response pathways, thereby contributing to cellular homeostasis and preconditioning responses.

At the molecular level, this adaptive response is largely mediated by activation of the nuclear factor erythroid 2-related factor 2 (Nrf2) pathway [43,44]. Low levels of ROS promote Nrf2 stabilization and nuclear translocation, leading to transcriptional activation of antioxidant response element (ARE)-driven genes, including those encoding detoxifying enzymes and redox-regulating proteins. This mechanism enables cells to buffer oxidative stress efficiently and maintain ROS levels below a critical threshold [45,46,47,48].

The transition from redox balance to oxidative stress represents a critical inflection point in LDRR responses. Once ROS generation exceeds the buffering capacity of antioxidant systems, oxidative stress increases sharply rather than gradually. This threshold-dependent transition is associated with mitochondrial dysfunction, lipid peroxidation, and activation of pro-apoptotic signaling pathways. Importantly, mitochondria play a central role in this process, acting both as a source and target of ROS, thereby amplifying oxidative damage through feed-forward mechanisms [20,49]. These observations indicate that oxidative stress under LDRR conditions is governed by a dynamic equilibrium rather than a linear accumulation process. Consequently, biological outcomes are determined not simply by radiation dose, but by the capacity of cellular systems to maintain redox homeostasis, providing a mechanistic basis for the nonlinear dose–response relationships observed in LDRR biology [20,50].

Mitochondrial dynamics further shape this threshold-dependent regulation. Processes such as mitochondrial fission, fusion, and mitophagy determine the balance between ROS production and clearance, thereby influencing cellular sensitivity to radiation-induced stress [51,52,53]. Dysfunctional mitochondria can act as a sustained source of ROS, amplifying oxidative signals and contributing to the transition from adaptive redox signaling to pathological oxidative stress [54,55,56].

Beyond mitochondrial ROS production, additional sources of oxidative stress further contribute to the nonlinear dynamics observed under LDRR conditions [57]. Radiation-induced damage to cellular membranes can initiate lipid peroxidation cascades, generating reactive aldehydes that propagate oxidative signaling [58,59]. Similarly, oxidative modifications of DNA and proteins can disrupt cellular signaling pathways and lead to persistent alterations in cellular function [60,61]. These processes are interconnected through feedback mechanisms that reinforce oxidative stress once a critical threshold is exceeded. For example, mitochondrial dysfunction can increase ROS production, which in turn exacerbates mitochondrial damage, creating a self-sustaining cycle of oxidative injury.

Importantly, ROS signaling under LDRR conditions also exhibits subcellular spatial heterogeneity. Localized ROS generation within mitochondria, the endoplasmic reticulum, or plasma membrane-associated complexes may differentially regulate signaling pathways, leading to compartment-specific responses within individual cells. This spatial dimension further contributes to the nonlinear nature of LDRR-induced oxidative responses and highlights the complexity of intracellular redox signaling.

## 4. Nonlinear Immune Modulation Under LDRR

Immune responses play a central role in mediating the biological effects of radiation exposure and are closely integrated with redox regulation [15,17,22]. Unlike oxidative stress, which follows a threshold-dependent pattern, immune responses to LDRR exhibit a nonlinear, often biphasic trajectory, reflecting complex regulatory mechanisms that integrate redox signaling with immune activation [34,35] (Cao et al. unpublished data). At low and intermediate dose rates (approximately 0.39 and 1.29 mGy/h within the studied range), LDRR is associated with activation of innate immune responses, including increased recruitment of neutrophils and macrophages, as well as elevated production of pro-inflammatory cytokines, such as tumor necrosis factor-α (TNF-α) and interleukin-1β (IL-1β) [15,17,22]. In contrast, at the higher end of the studied range (3.46 mGy/h), immune responses shift toward attenuation, characterized by systemic lymphopenia and suppressed inflammatory signaling. These responses are largely driven by redox-sensitive signaling pathways, including nuclear factor kappa B (NF-κB) and activator protein-1 (AP-1), which are activated by ROS-mediated stress signals. The activation of these transcription factors leads to the expression of genes involved in inflammation, immune cell recruitment, and tissue remodeling [17,21]. It should be noted that the specific activation mechanisms of NF-κB and AP-1 described here have been characterized primarily in acute or high-dose radiation models; their operation under chronic LDRR conditions is inferred from ROS-dependent signaling kinetics and is consistent with available experimental data, but direct mechanistic validation under LDRR-specific conditions remains limited.

Radiation-induced DNA damage further contributes to immune activation by releasing nuclear and mitochondrial DNA into the cytosol, thereby activating the cGAS-STING pathway [62,63,64]. Although the cGAS–STING pathway has been characterized primarily in the context of high-dose radiation-induced DNA damage, it is inferred that under LDRR conditions, moderate and sustained pathway activation may enhance immune surveillance by promoting type I interferon responses. This inference is consistent with dose rate-dependent DNA damage kinetics but awaits direct experimental validation under chronic LDRR conditions. However, at higher dose rates within the LDRR range, immune responses often shift toward attenuation or adaptation. This transition likely reflects the activation of regulatory pathways that limit excessive inflammation, including the production of anti-inflammatory cytokines, the activation of immune checkpoints, and cellular exhaustion. Prolonged or excessive activation of inflammatory signaling pathways can lead to tissue damage, necessitating feedback inhibition to restore homeostasis [62,63].

These observations indicate that immune responses under LDRR are tightly regulated and context-dependent rather than stochastic. The biphasic nature of immune modulation suggests an optimal exposure window in which immune activation is beneficial, whereas deviations from this range may lead to suppression or pathological inflammation. This nonlinear behavior underscores the importance of integrating both dose rate and biological context when interpreting radiation-induced immune responses.

Beyond innate immune activation, LDRR also influences adaptive immune responses, including T-cell activation, differentiation, and long-term immune surveillance [15,65,66]. The following observations are largely inferred from general radiation immunology; direct evidence for these adaptive immune effects under chronic LDRR conditions specifically remains sparse. Activation of pathways such as cGAS-STING and NF-κB shapes antigen presentation and cytokine environments that regulate T-cell responses [67,68,69]. Under LDRR conditions, these processes may enhance immune surveillance and promote controlled immune activation, whereas dysregulation at higher exposure levels may contribute to immune suppression or exhaustion [17,66].

Importantly, immune responses under LDRR should be viewed as components of an integrated regulatory network rather than isolated signaling events [19]. Cross-talk between innate and adaptive immune systems links early inflammatory responses to long-term immune surveillance, while regulatory mechanisms, including the activation of regulatory T cells and the expression of immune checkpoint molecules, attenuate immune responses at higher dose rates [70]. Although these processes limit excessive inflammation, they may also compromise effective immune surveillance under certain conditions. This dynamic balance further reinforces the concept of a tightly controlled, nonlinear landscape of the immune response [71].

In addition to activation and suppression phases, the resolution of inflammation represents a critical but often overlooked component of immune responses under LDRR conditions. Proper resolution is essential for restoring tissue homeostasis and preventing chronic inflammation. Dysregulation of this process may lead to prolonged immune activation or incomplete recovery, further complicating the biological outcomes of LDRR exposure. Incorporating both the initiation and resolution phases of immune responses into LDRR models will therefore be essential for developing a comprehensive understanding of radiation-induced immune modulation.

## 5. Compartment-Specific Sensitivity to Radiation

A defining feature of LDRR responses is their pronounced compartment-specificity. Biological responses to radiation vary significantly across tissues and fluid compartments, reflecting differences in local microenvironmental conditions, including redox balance, immune composition, and metabolic activity [14,72]. Different tissues exhibit varying baseline levels of oxidative stress and antioxidant capacity, which influence their sensitivity to radiation-induced ROS. For example, metabolically active tissues such as the liver and muscle may be more susceptible to redox imbalance. In contrast, tissues with robust antioxidant defenses may be more resistant to oxidative damage [50,73]. Similarly, immune-rich compartments may respond more readily to radiation-induced signals due to the presence of resident immune cells and cytokine networks [74,75,76]. Differences also influence the compartment-specific nature of radiation responses in vascularization, oxygen availability, and cellular turnover rates. Hypoxic environments, for instance, may alter ROS generation and signaling pathways, leading to distinct biological outcomes compared to well-oxygenated tissues [77,78]. Additionally, tissue-specific expression of ion channels and signaling molecules can further modulate responses to radiation [79].

Beyond these observations, the interplay between oxidative stress and immune signaling emerges as a critical determinant of compartment-specific responses. The same radiation exposure can produce divergent outcomes depending on how redox signaling interacts with local immune regulation within a given microenvironment [74]. This highlights the importance of considering spatial and physiological context when interpreting radiation effects. These findings suggest that systemic measurements alone may not adequately capture localized biological responses, underscoring the need for multi-compartment analysis. Understanding compartment-specific sensitivity is therefore essential for developing accurate biomarkers and targeted therapeutic strategies.

The following section and table integrate findings from the author’s experimental program, spanning blood/peritoneal, reproductive, and joint compartments, with the independently published literature. The author’s experimental data are explicitly positioned as direct extensions of established patterns of compartment-specific radiation sensitivity, and are not intended as primary evidence for the conceptual framework. Independent corroboration for each compartment is described below. The compartment-specific biological responses described above are summarized in Table 2, highlighting the distinct dose–response patterns, nonlinear (blood/PLF), biphasic (synovial tissue), threshold-dependent (testis), and minimal (BALF), observed across compartments under identical LDRR conditions. These contrasting patterns underscore the importance of compartment-specific analysis and form the empirical basis for the integrated framework proposed in Section 6. Preliminary data from synovial fluid under identical LDRR conditions suggest a similar biphasic immune trajectory, though these findings await independent validation (Cao et al., unpublished data). 

## 6. Integrated Model: Redox–Immune Coupling Under LDRR

**Figure 1:**
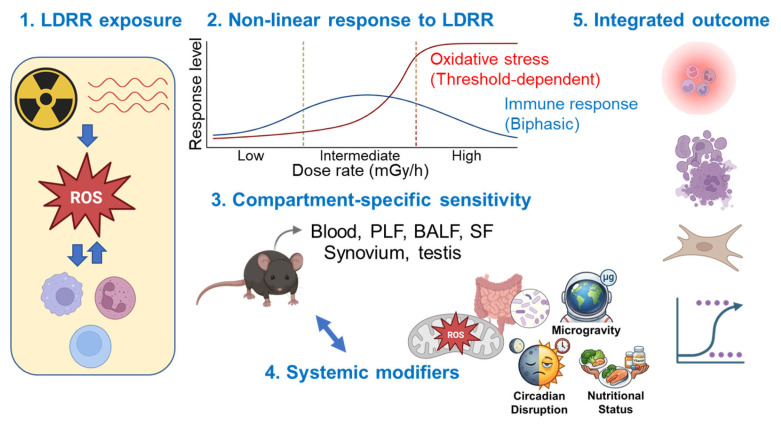
Conceptual model of nonlinear redox–immune coupling under low-dose-rate radiation (LDRR). The model integrates five components illustrated from left to right: (1) reactive oxygen species (ROS) generation as the primary mediator; (2) threshold-dependent oxidative stress and biphasic immune dynamics; (3) compartment-specific sensitivity across body fluids, synovial tissue, and reproductive organs; (4) systemic modifiers including mitochondrial function, nutritional status, and microbiome; and (5) downstream biological outcomes including inflammation, oxidative damage, and tissue remodeling. Mechanistic details are described in the main text (Section 3, Section 4, Section 5 and Section 6). In Panel 2, the green and red dashed vertical lines denote putative transition thresholds for immune and oxidative responses, respectively. The dotted symbols and sigmoidal curve in Panel 5 illustrate nonlinear and threshold-dependent integrated biological outcomes. Downward arrows indicate progression of biological events, and bidirectional arrows indicate reciprocal interactions between ROS and immune signaling or between local compartmental responses and systemic modifiers. Note: PLF, peritoneal lavage fluid; BALF, bronchoalveolar lavage fluid; SF, synovial fluid.

The integrated model proposed here is conceptually illustrated in Figure 1. LDRR exposure generates ROS in a dose rate-dependent manner, driving nonlinear biological responses, threshold-dependent oxidative stress and biphasic immune modulation, that vary across biological compartments (blood, PLF, BALF, synovial fluid, and testis) and are further shaped by systemic modifiers including circadian disruption, nutritional status, and microgravity. Within this framework, oxidative stress and immune responses are functionally coupled but exhibit distinct, nonlinear dynamics under LDRR.

At low dose rates, mild ROS generation promotes adaptive signaling pathways, including Nrf2 activation, while simultaneously stimulating controlled immune responses [45,47]. In this phase, redox homeostasis is maintained, and immune activation contributes to surveillance and tissue repair. At intermediate dose rates, increased ROS production challenges antioxidant defenses, leading to metabolic stress and amplification of inflammatory signaling pathways [34,35] (Cao et al. unpublished data). This transitional phase reflects a balance between adaptation and dysfunction, with both protective and detrimental processes occurring simultaneously.

**Figure 2:**
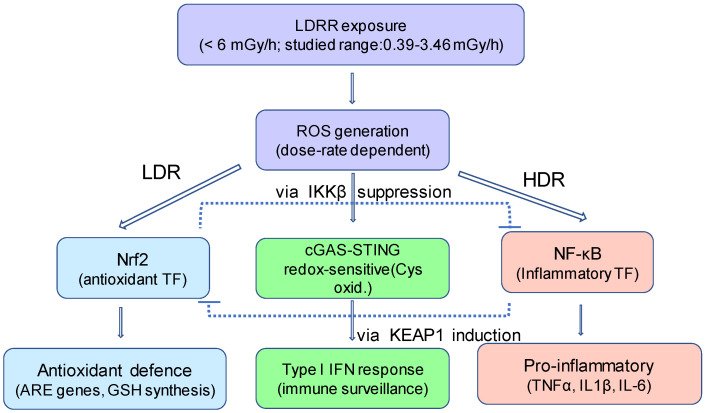
LDRR exposure (studied range: 0.39–3.46 mGy/h; within the ICRP-defined threshold of <6 mGy/h) generates reactive oxygen species (ROS) in a dose rate-dependent manner. At low dose rates (LDR), moderate ROS preferentially activate Nrf2 (antioxidant transcription factor), driving antioxidant response element (ARE)-dependent gene expression and glutathione (GSH) synthesis. At high dose rates, excessive ROS activate NF-κB (inflammatory transcription factor), promoting pro-inflammatory cytokine production (TNF-α, IL-1β, IL-6). The cGAS–STING pathway functions as a redox-sensitive intermediary: moderate ROS promote pathway engagement and Type I interferon (IFN) responses supporting immune surveillance, while excessive oxidation of cysteine residues (Cys oxid.) impairs pathway activity. Blunt-ended dotted lines indicate inhibitory cross-talk between Nrf2 and NF-κB; solid arrows indicate activation or downstream signaling pathways. Note: IKKβ, IκB kinase β; KEAP1, Kelch-like ECH-associated protein 1; TF, transcription factor, Nrf2, nuclear factor erythroid 2-related factor 2; NF-κB, nuclear factor kappa-light-chain-enhancer of activated B cells; cGAS-STING, cyclic GMP–AMP synthase (cGAS)-stimulator of interferon genes (STING).

At higher dose rates within the LDRR range, oxidative stress exceeds the buffering capacity of cellular systems, resulting in disruption of redox homeostasis, immune attenuation, and tissue-level outcomes including apoptosis, fibrosis, and functional impairment [34,35,90] (Cao et al. unpublished data). A central feature of this model is the dynamic molecular cross-talk between NF-κB-mediated inflammatory signaling and Nrf2-driven antioxidant responses (Figure 2) [91,92]: Nrf2 activation suppresses NF-κB-driven transcription by upregulating ARE genes whose products limit ROS availability and sequester IκB kinase β (IKKβ) activity [91,93]; conversely, NF-κB represses Nrf2 by recruiting HDAC3 to the Nrf2 promoter and inducing Kelch-like ECH-associated protein 1 (KEAP1) expression [94,95]. Additionally, the cGAS-STING pathway exhibits direct redox sensitivity, whereby oxidative modification of cysteine residues modulates pathway activity in an ROS concentration-dependent manner, providing a direct molecular link between the redox environment and innate immune activation [91,96]. It should be noted that these molecular interactions have been characterized primarily in non-LDRR studies; their operation under chronic LDRR conditions is inferred from dose rate-dependent ROS kinetics and requires direct experimental validation.

Building on these molecular mechanisms, the dose rate-dependent ROS regulation is inferred to generate a sequential immune trajectory across the three studied dose rates. At the lowest dose rate studied (0.39 mGy/h), the observed pattern of immune cell redistribution and mild oxidative stress markers is consistent with preferential Nrf2 stabilization suppressing NF-κB activity and promoting an adaptive immune phenotype [45,47], though direct measurement of these transcription factors was not performed in the present experimental system. At the intermediate dose rate (1.29 mGy/h), the documented metabolic stress and inflammatory marker elevation suggests co-activation of both Nrf2 and NF-κB, producing a transient inflammatory peak consistent with the immune activation phase documented in PLF [34], and preliminarily observed in synovial fluid (Cao et al., unpublished data). At the highest dose rate studied (3.46 mGy/h), the observed systemic lymphopenia and suppressed inflammatory markers are consistent with NF-κB-mediated KEAP1 induction collapsing the adaptive response [68,91], and possible over-oxidation of cGAS and STING impairing innate immune signaling [63,64], collectively producing the immune attenuation, though these mechanistic interpretations remain to be directly validated. This ROS-gated sequential activation model mechanistically couples the redox and immune axes [1,97], generating the compartment-specific biphasic output that distinguishes the LDRR response from monotonic LNT predictions [29,98].

These dose rate windows represent a conceptual framework extrapolated from available experimental data at three discrete dose rates (0.39, 1.29, and 3.46 mGy/h); the precise phase boundaries are not yet empirically established and likely vary with tissue type, species, and exposure duration. These mechanistic interpretations therefore require direct experimental validation, including transcription factor activity assays and pathway-specific markers, under chronic LDRR conditions. Other experimental studies have employed dose rates outside this specific range; where directly comparable data exist, these are incorporated to provide broader context [7,11,29,36,86,87].

## 7. Clinical and Translational Implications

The nonlinear and compartment-specific nature of LDRR responses has important implications for clinical practice and translational research. In radiotherapy, surrounding normal tissues are frequently exposed to low-dose radiation, often referred to as the low-dose bath, which may contribute to long-term toxicity and secondary complications [37,99,100]. These effects are unlikely to be explained by cumulative dose alone and instead may reflect the nonlinear and context-dependent nature of LDRR responses. Understanding how oxidative stress and immune responses are dynamically regulated under LDRR conditions is, therefore, critical for improving treatment outcomes and minimizing adverse effects.

One key implication of this framework is the need for more precise biomarkers that capture localized and context-dependent radiation responses. Traditional systemic biomarkers may not adequately reflect compartment-specific changes, particularly in tissues with distinct metabolic and immune characteristics. In this regard, biological fluids such as blood, PLF, and BALF offer promising platforms for monitoring radiation-induced alterations in redox balance and immune activity. Specific candidate biomarkers derivable from the proposed framework include: (1) oxidative stress markers, glutathione oxidation ratio (GSH/GSSG) [101], 8-hydroxy-2′-deoxyguanosine (8-OHdG) [71,102], and plasma 4-hydroxynonenal (4-HNE) [102], to capture threshold-dependent redox dynamics; (2) immune phase indicators, circulating cytokine panels (IL-6, TNF-α, IL-10, TGF-β1) [34,71] and immune cell subset ratios (CD4+ Treg/Teff, neutrophil-to-lymphocyte ratio) [70], to discriminate the activation from the attenuation phase; and (3) compartment-specific signatures reflecting local redox and immune microenvironments [34,103]. Testable hypotheses include: (a) the GSH/GSSG ratio will show a nonlinear decline with a sharp inflection at the redox threshold; (b) the serum IL-10/TNF-α ratio will peak at intermediate dose rates and decline at higher exposure levels; and (c) PLF and synovial fluid biomarker profiles will show greater sensitivity and specificity for LDRR phase discrimination than BALF.

The nonlinear nature of LDRR responses also has significant implications for therapeutic intervention. Antioxidant strategies may exert differential effects depending on the exposure phase. While antioxidant supplementation may be beneficial at higher doses by reducing excessive oxidative stress, it may interfere with adaptive signaling mechanisms at lower doses [36,99,104]. Similarly, anti-inflammatory interventions must be carefully timed, as early immune activation may support tissue repair and tumor control, whereas prolonged or dysregulated inflammation may promote fibrosis and functional decline [105,106].

Emerging evidence further suggests that persistent low-dose exposure can induce subtle but sustained alterations in tissue homeostasis, including mitochondrial dysfunction, low-grade inflammation, and dysregulated immune surveillance. These changes may accumulate over time and contribute to delayed tissue injury, particularly in radiosensitive organs, even in the absence of overt cytotoxic damage [97,107]. Incorporating LDRR-specific biological mechanisms into radiotherapy planning and long-term risk assessment is therefore increasingly important.

From a broader perspective, these findings support a shift toward biologically informed radiotherapy paradigms. Variability in patient-specific factors, including metabolic status, immune competence, and microbiome composition, may significantly influence radiation sensitivity and treatment outcomes [108,109,110,111]. Integrating these variables into clinical decision-making could improve therapeutic precision and reduce adverse effects. This approach aligns with the broader movement toward personalized medicine and underscores the need for integrated models that account for both physical and biological determinants of radiation response.

In addition to these considerations, the clinical relevance of LDRR responses becomes particularly evident in specific treatment scenarios. For example, in intensity-modulated radiotherapy (IMRT) and proton therapy, heterogeneous low-dose fields are generated in surrounding normal tissues, creating spatially variable biological exposures [112,113,114,115]. These spatial dose variations may lead to region-specific shift in redox homeostasis and local immune signaling, which are not captured by conventional dose-volume metrics.

Furthermore, patient-specific factors such as age, pre-existing inflammation, and metabolic disorders may further modulate LDRR responses. For instance, tissues with elevated baseline oxidative stress or impaired antioxidant capacity may exhibit heightened sensitivity to LDRR-induced damage. In contrast, variations in immune competence may influence the balance between protective immune activation and detrimental inflammation [23,99,116,117]. These considerations highlight the need for patient-stratified approaches when evaluating radiation-induced risks and therapeutic outcomes.

Collectively, these observations underscore that effective translation of LDRR biology into clinical practice will require a shift from uniform treatment paradigms toward context-aware and patient-specific strategies that integrate both physical dose distribution and biological response dynamics. To further illustrate how these principles translate into clinical practice, specific treatment scenarios in which LDRR effects are particularly relevant are discussed below.

### Clinical Scenarios Requiring LDRR-Specific Consideration

In clinical radiotherapy, several treatment scenarios highlight the need to explicitly consider the biological effects of LDR exposure beyond conventional dose-based evaluation. For example, in IMRT and volumetric modulated arc therapy (VMAT), optimization algorithms prioritize tumor conformity, often at the cost of increasing the volume of normal tissue exposed to low-dose radiation [118,119]. Although these dose distributions are considered acceptable within current planning constraints, they may induce biologically relevant changes in redox balance and immune activity that are not reflected in standard dose–volume histograms. Importantly, such responses may arise from threshold-dependent redox regulation and nonlinear immune modulation, as proposed in this review.

Similarly, in proton therapy, reducing high-dose exposure is often accompanied by an expanded low-dose distribution due to secondary particle production and beam modulation [120,121]. These exposure patterns raise important questions about long-term biological effects, particularly in tissues repeatedly exposed to sub-threshold oxidative stress [101,114]. In this context, repeated exposure near the redox threshold may result in cumulative shifts in cellular homeostasis, potentially altering the balance between adaptive signaling and pathological responses. Such conditions may not result in immediate toxicity but could contribute to delayed functional alterations over time.

Another clinically relevant scenario involves fractionated radiotherapy, where repeated low-dose exposures may interact with ongoing cellular repair and immune processes [17,122]. Under these conditions, the temporal spacing of radiation delivery may influence whether biological responses remain within an adaptive range or transition beyond critical thresholds into pathological states. This is particularly relevant for immune responses, which follow a biphasic trajectory and may shift from protective activation to suppression or dysregulation depending on exposure patterns. These observations suggest that treatment scheduling, in addition to total dose, may play a critical role in determining clinical outcomes.

It should be noted that fractionated external beam radiotherapy (EBRT) typically does not constitute low-dose-rate radiation (LDRR) in the strict dosimetric sense, as each fraction is delivered at dose rates orders of magnitude higher than the LDRR range (typically 2–6 Gy/min, reaching up to 24 Gy/min with FFF beams) [9]. For standard RT, the LDRR-relevant component is the cumulative low-dose bath received by surrounding normal tissue, rather than the high-dose delivery to the target volume. The biological implications discussed here pertain specifically to this low-dose normal tissue exposure and should not be interpreted as applying to the fractionated delivery paradigm as a whole. This distinction also applies to the proton therapy and IMRT/VMAT scenarios: LDRR effects are relevant to the low-dose peripheral bath, not to the target-volume dose delivery (note: A notable exception is Pulsed Low-Dose Rate Radiotherapy (PLDR), which specifically targets the tumor volume using LDRR principles) [9].

Taken together, these examples illustrate that LDRR effects are not merely theoretical considerations but are embedded within routine clinical practice. Incorporating LDRR-specific biological insights, particularly those related to threshold-dependent redox regulation and nonlinear immune dynamics, into treatment planning and evaluation may improve the prediction of normal tissue responses and support more biologically informed radiotherapy strategies. 

Three clinical and occupational scenarios illustrate the direct translational relevance of the LDRR framework.

1. In LDR brachytherapy for prostate cancer (^125^I permanent seeds), the initial dose rate is approximately 7.0 cGy/h (ABS recommendations) providing [123,124], a continuous protracted exposure that directly mirrors the LDRR conditions described in this review. The framework predicts sub-threshold to threshold-level ROS generation in periurethral tissue, with potential biphasic immune modulation and delayed fibrosis risk via progressive collagen deposition. Candidate biomarkers include GSH/GSSG, 8-OHdG, neutrophil-to-lymphocyte ratio (NLR), and IL-6/TGF-β1, though these required prospective validation.
2. In radionuclide therapy (^131^I, ^177^Lu-PSMA, ^223^Ra) [125,126], normal organ dose rates frequently fall within the 0.1–5 mGy/h range, making compartment-specific monitoring directly relevant. Clinically documented responses include hematopoietic suppression in the bone marrow, oxidative stress in thyroid and salivary glands, and reproductive toxicity risk in the testis above specific dose rate thresholds [125,126]. Candidate monitoring biomarkers include CBC with differential (RET, WBC, P-LCR), salivary amylase, and testosterone/FSH levels.
3. In occupational and spaceflight settings, chronic LDRR at 0.001–1 mGy/h represents the lower end of the redox threshold range. However, extreme conditions such as Solar Particle Events (SEPs) can cause dose rates to spike to approximately 57 mGy/h, which significantly exceeds the baseline exposure. Such environments lead to potential long-term immune drift, cumulative sub-threshold oxidative signaling, and compartment-specific adaptation [29,127]. Longitudinal monitoring using cytokine panels, periodic GSH/GSSG measurements, and NLR trends may provide early indicators of cumulative biological effects in these chronically exposed populations.

Across all scenarios, the framework predicts that biological responses are governed not merely by total dose but by the dose rate-dependent balance between redox threshold activation and immune phase transitions.

## 8. Integrated Perspective and Future Directions

The collective evidence reviewed here supports a shift from a dose-centric interpretation of radiation biology toward a systems-level framework, in which biological outcomes emerge from the dynamic interplay between oxidative stress and immune regulation. Under LDRR conditions, these responses do not scale linearly with exposure but instead reflect nonlinear dynamics shaped by threshold-dependent redox signaling and context-dependent immune modulation [1,39,128]. This perspective reconciles previously inconsistent findings by positioning LDRR responses as regulated biological processes rather than stochastic events.

A defining feature of this framework is the differential dose–response behavior of oxidative stress and immune signaling. While oxidative stress is governed by a threshold-dependent transition from adaptive redox regulation to pathological imbalance, immune responses exhibit a biphasic trajectory characterized by activation at lower exposure levels and attenuation or adaptation at higher levels. Importantly, these processes are not independent but are functionally coupled through shared signaling networks, including redox-sensitive transcription factors and inflammatory pathways [27,129,130,131]. This coupling provides a mechanistic basis for the nonlinear and context-dependent nature of LDRR responses.

Equally important is the recognition that radiation-induced effects are highly compartment-specific. Biological tissues and fluid environments differ substantially in their baseline redox state, immune composition, and metabolic activity, leading to distinct response patterns under comparable exposure conditions [15,34,132,133,134]. As a result, radiation effects cannot be accurately predicted solely from dose but must be interpreted within the context of local microenvironments. This insight highlights the limitations of traditional reductionist approaches and underscores the need for integrative models that incorporate spatial heterogeneity.

In addition to local microenvironmental factors, systemic modifiers, including metabolic status, microbiome composition, and environmental stressors, further shape LDRR responses. These factors influence both redox balance and immune activity, contributing to the variability observed across experimental systems and physiological conditions [20,135,136,137]. Viewing radiation exposure within this broader physiological context provides a more coherent framework for understanding how multiple regulatory inputs converge to determine biological outcomes.

Looking forward, advancing LDRR research will require approaches capable of capturing both spatial and temporal complexity. High-resolution technologies such as single-cell and spatial transcriptomics, as well as proteomic profiling, offer powerful tools to resolve cell-type-specific redox and immune dynamics within tissues [138,139,140]. These methods will be critical for identifying cellular subpopulations that exhibit differential sensitivity to LDRR-induced stress and for mapping localized responses within heterogeneous microenvironments.

Moreover, time-resolved studies will be essential for defining the progression of LDRR-induced responses, particularly the transition from adaptive signaling to pathological states [141,142]. Such temporal analyses will help clarify the conditions under which protective mechanisms fail and may inform the development of phase-specific intervention strategies. In this context, bridging temporal dynamics with mechanistic insights will be key to improving predictive accuracy.

Finally, integrating experimental findings with computational modeling and systems biology frameworks will be crucial for translating complex, nonlinear interactions into predictive, clinically relevant models [103,143]. By incorporating multiple layers of biological information, including redox status, immune signaling, metabolic state, and environmental modifiers, these integrative approaches may enable personalized predictions of radiation sensitivity and support the optimization of therapeutic interventions. Taken together, these advances highlight that a comprehensive understanding of LDRR responses requires integrating mechanistic insight with systems-level analysis. This paradigm is essential not only for resolving current inconsistencies in radiation biology but also for enabling precise and context-aware applications in radiotherapy, environmental exposure assessment, and human health. An improved understanding of these processes may also inform regulatory frameworks and public health strategies related to chronic low-dose radiation exposure.

## 9. Strengths and Limitations

This narrative review presents several notable strengths. First, it integrates evidence from multiple biological compartments and experimental systems, enabling a systems-level synthesis rarely attempted in the LDRR literature. Second, the proposed redox–immune coupling framework explicitly articulates molecular mechanisms (Nrf2/NF-κB reciprocal regulation; cGAS-STING redox sensitivity) that link the two principal response axes, providing a testable mechanistic model rather than a descriptive overview. Third, the clinical translation section explicitly connects biological mechanisms to specific radiotherapy scenarios, including IMRT, proton therapy, and fractionated regimens. Alongside these strengths, this review also has several limitations. First, as a narrative review, the evidence synthesis is subject to selection bias; findings may not fully represent the complete landscape of LDRR research. Second, a substantial portion of the compartment-specific mechanistic evidence derives from a limited number of experimental systems, primarily in murine models, and direct translation to human physiology requires further validation. Third, several supporting studies originate from the same research group, which limits generalizability pending independent replication. Fourth, the precise dose rate thresholds proposed (0.39–3.46 mGy/h) are based on available experimental data and likely vary with tissue type, species, and genetic background. Fifth, many mechanistic studies cited were conducted using acute or in vitro models, which may not fully recapitulate chronic LDRR conditions in vivo. Finally, the field is rapidly evolving, and some findings cited here may require reassessment as new evidence emerges.

### Limitations of the Proposed Framework

Beyond the general study limitations noted above, several inherent limitations of the redox–immune coupling framework itself deserve consideration. First, the framework predicts threshold-dependent and biphasic responses based primarily on murine experimental data; the specific threshold values, molecular parameters, and tissue-specific modifiers described here may not directly translate to human physiological conditions or clinical dose rate ranges, and prospective validation in human cohorts is required before any clinical application can be considered. Second, and most critically for therapeutic translation, the framework generates paradoxical implications for interventional strategies. During the adaptive phase of LDRR (low dose rates), moderate ROS are required to drive beneficial Nrf2 activation and immune surveillance. In this context, antioxidant supplementation could paradoxically suppress the protective adaptive response rather than enhance it. Similarly, anti-inflammatory agents (e.g., NSAIDs, corticosteroids, cytokine inhibitors) administered during the immune activation phase may blunt biologically beneficial immune modulation, potentially worsening outcomes. Conversely, the same interventions may confer protection during the pathological high-dose-rate phase. This context dependency means that therapeutic recommendations cannot be uniformly applied; their efficacy and safety depend critically on dose rate, timing, and compartment—parameters that cannot yet be reliably determined in routine clinical practice. Third, all clinical implications proposed in this review (biomarker development, radiotherapy optimization, therapeutic strategies) remain speculative and are not supported by prospective clinical data. The framework should be regarded as a hypothesis-generating tool providing mechanistic rationale for future investigation, not as a basis for current clinical recommendations.

## 10. Conclusions

LDRR induces nonlinear biological responses characterized by threshold-dependent oxidative stress and biphasic immune modulation. These responses are highly dependent on biological compartment and systemic context, reflecting the integration of multiple regulatory inputs rather than simple dose accumulation. The present review proposes a mechanistic hypothesis that may help account for previously inconsistent findings across experimental systems, pending further experimental and clinical validation.

Importantly, this perspective has potential implications for research and, if validated in human studies, for clinical practice. Incorporating the nonlinear and context-dependent nature of LDRR responses into experimental design and biomarker development may, if the framework is validated, contribute to improving the prediction of normal tissue outcomes. However, direct application to radiotherapy planning or therapeutic strategies requires prospective clinical evidence and should be approached with caution, given the context-dependent and potentially paradoxical effects of interventions described in Section 9.

Future efforts should focus on integrating mechanistic insights with high-resolution and systems-level approaches to define better spatial, temporal, and patient-specific variability in radiation responses. Such advances will be essential for translating LDRR biology into clinically actionable frameworks and for optimizing the safe and effective use of radiation in diverse settings.

## Figures and Tables

**Table 1:** Summary of key experimental studies reporting dose rate, exposure conditions, and biological effect magnitude under low-dose-rate radiation.

Study	Model/Species	Radiation Type	Dose Rate	Cumulative Dose	Duration	Biological System	Key Findings (Molecular/Cellular)	Response Pattern
Kim et al. (2026) [34]	C57BL/6 mice (male)	^137^Cs gamma, continuous	0.39, 1.29, 3.46 mGy/h	0.19, 0.64, 1.72 Gy	21 days	Blood, PLF, BALF	↑ ALT, ↑ AST (blood, 0.39 mGy/h); ↑ AST, ↑ CK, ↑ LAC (PLF, 1.29 mGy/h); ↑ ALPIF (PLF, 0.39 mGy/h) ↑ NEUT (blood, 0.39); ↓ WBC, ↓ LYMPH (blood, 3.46); ↑ PMNC, ↓ LYMPH (PLF, 0.39–1.29); ↑ RET, ↓ P-LCR (blood, 3.46); BALF: no change	Nonlinear, compartment-specific; biphasic hematological response
Kim et al. (2025) [35]	C57BL/6 mice (male)	^137^Cs gamma, continuous	0.39, 1.29, 3.46 mGy/h	0.19, 0.64, 1.72 Gy	21 days	Testis, epididymis	↑ ROS (3.46 mGy/h only); ↑ collagen dose-dependently from 0.39 mGy/h; ↑ α-SMA, PDGFRα mRNA (3.46 mGy/h) ↑ TUNEL+ cells (1.29, 3.46 mGy/h); ↑ comet tail DNA (3.46); ↓ spermatogenic cell count (3.46)	Two-phase: progressive fibrosis; threshold-dependent apoptosis/ROS
Goldberg et al. (2006) [37]	Humans (*n* = 8)	LDR photons	Acute (~2–6)	1, 10, 100 cGy	Single session	Skin (Punch biopsy)	Biosignature: Akt/PI3K, TGF-β signaling, keratins, and zinc finger proteins	Significant linear response
Vieira Dias et al. (2018) [36]	HAoECs	^60^Co gamma	6 mGy/h vs 1 Gy/min	0.05–2.0 Gy	2 min, 16 days	Vascular endothelial cells	↑ SOD-1, SOD-2, CAT; ↑ VEGF, VEGF-R2, eNOS; ↑ TGF-β1, ↓ IL-6	Threshold (0.5–1.0 Gy) for loss of function; radioadaptive response at 2.0 Gy LDR
Lowe et al. (2022) [29]	Review (multiple species)	Multiple	<0.1 mGy/min	Variable	Variable	Multi-organ	LDR reduces inflammation; increases lifespan via adaptation	Nonlinear: inverse dose rate effect for mutation and cataracts
Liu et al. (2020) [38]	Balb/c mice (male)	^60^Co gamma	3.93 cGy/min (protracted)	0.2–1.0 Gy	10 times for 6 weeks	Spleen, Thymus, Blood	↓ NK cells, macrophages, DCs (day 2); ↑ Nrf2, HO-1, iNOS (day 14)	Biphasic cytokine response (IFN-γ, IL-12, IL-4); recovery after day 7
Dahl et al. (2021) [5]	CBA/CaOlaHsd, C57BL/6NHsd (male)	^60^Co gamma, chronic	2.5-100 mGy/h	3.0 Gy	1.25–50 days	Liver (hepatic)	perturbed pathways; lipid metabolism and inflammation; no DNA methylation	Nonlinear; dose rate and strain-specific transcriptional responses

Note: PLF, peritoneal lavage fluid; BALF, bronchoalveolar lavage fluid; HAoECs, Human Aortic endothelial cells; LTBI, low-dose total body irradiation; ROS, reactive oxygen species; Nrf2, nuclear factor erythroid 2-related factor 2; NEUT, neutrophils; LYMPH, lymphocytes; PMNC, polymorphonuclear cells; WBC, white blood cells; RET, reticulocytes; P-LCR, platelet–large cell ratio; AST, aspartate aminotransferase; ALT, alanine aminotransferase; CK, creatine kinase; LAC, lactate; ALPIF, alkaline phosphatase isoenzyme fraction; α-SMA, alpha-smooth muscle actin; PDGFRα, platelet-derived growth factor receptor alpha; TUNEL, terminal deoxynucleotidyl transferase dUTP nick end labeling; HO-1, heme oxygenase-1; AKT, protein kinase B; PI3K, phosphoinositide-3-kinase; CAT, Catalase; NK, natural killer; DC, Dendritic cell; i(e)NOS, inducible (endothelial) nitric oxide synthase; SOD, superoxide dismutase; VEGF, vascular endothelial growth fctor. ↑, increased; ↓, decreased relative to the corresponding control or reference group.

**Table 2:** Summary of compartment-specific biological responses to low-dose-rate radiation.

Biological Compartment	Key Findings	Dose–Response Pattern	Dominant Mechanism	Clinical Implication	Representative References
Blood/PLF	Immune cell redistribution (↑ neutrophils, ↓ lymphocytes) metabolic changes	Nonlinear	Adaptive immune activation	Biomarker potential	[34,80,81,82,83]
Synovial fluid/ tissue	↑ IL-4, ↓ IL-17A A shift toward an anti-inflammatory milieu after LDRT	Biphasic	Local immune regulation Inflammatory musculoskeletal disorders	Pain reduction, Biomarker potential For early inflammatory response	[84,85]
Testis	Apoptosis, fibrosis, and ROS increase above the high dose	Threshold- dependent	Oxidative stress- driven damage	Reproductive toxicity	[7,35,86,87]
BALF	Minimal to no significant change in cellular or protein markers	Dose-rate dependent	Low sensitivity compartment	Limited Sensitivity for chronic LDRR monitoring	[34,88,89]

Note: PLF, peritoneal lavage fluid; BALF, bronchoalveolar lavage fluid; IL, interleukin; LDRT, low-dose radiotherapy. ↑, increased; ↓, decreased relative to the corresponding control or reference group.

## Data Availability

No new data were created or analyzed in this study.

## Abbreviations

The following abbreviations are used in this manuscript:

LDRR	Low-dose-rate radiation
ROS	Reactive oxygen species
PLF	Peritoneal lavage fluid
BALF	Bronchoalveolar lavage fluid
TNF-α	Tumor necrosis factor-alpha
IL-1β	Interleukin-1 beta
Nrf2	nuclear factor erythroid 2-related factor 2
NF-κB	nuclear factor kappa-light-chain-enhancer of activated B cells
cGAS	cyclic GMP–AMP synthase
STING	stimulator of interferon genes
